# Lobaric Acid Exhibits Anticancer Potential by Modulating the Wnt/β‐Catenin Signaling Pathway in MCF‐7 Cells

**DOI:** 10.1002/prp2.70142

**Published:** 2025-06-27

**Authors:** Şeyda Nur Kalın, Kübra Nur Bayındırlı, Emine Toraman, Şükran Günaydın, Fatmanur Keleş, Ahmet Altay, Harun Budak

**Affiliations:** ^1^ Department of Molecular Biology and Genetics Science Faculty, Atatürk University Erzurum Turkey; ^2^ Department of Chemistry Faculty of Science and Arts, Erzincan Binali Yıldırım University Erzincan Turkey; ^3^ Department of Laboratory and Veterinary Sciences Narman Vocational College, Atatürk University Erzurum Turkey; ^4^ Department of Molecular Biology and Genetics Faculty of Engineering and Natural Sciences, Kütahya Health Sciences University Kütahya Turkey

**Keywords:** apoptosis, breast cancer, cytotoxicity, expression, Wnt/β‐catenin pathway

## Abstract

Lichen secondary metabolites with many remarkable biological activities are used in cancer treatments due to their low side effects and high anticancer potential. In particular, these metabolites constitute an interesting research area in cancer treatments due to their potential to induce apoptosis and suppress metastasis by inhibiting cancer‐related signaling pathways. The Wnt/β‐catenin signaling pathway plays a role in important biological processes such as oncogenesis, cell cycle regulation, cell proliferation, metastasis, differentiation, apoptosis, and drug resistance. Therefore, inhibition of this pathway is a potential target in cancer therapies. There is no detailed study explaining the potential anticancer molecular mechanism of the lichen secondary metabolite lobaric acid (LA) on breast cancer. Here, it is aimed to investigate the effect of LA on viability, apoptosis, and migration in MCF‐7 cells and to elucidate the relationship between the potential anticancer effect and the Wnt/β‐catenin signaling pathway. The dose‐ and time‐dependent viability of LA‐treated MCF‐7 cells was evaluated by XTT assay, and the IC_50_ value was determined as 44.21 μg/mL at 48 h. LA increased the apoptotic cell population, as shown by flow cytometry analysis, qPCR, and Western blot results. LA inhibited β‐catenin by inducing GSK3‐β protein expression, thereby suppressing Wnt/β‐catenin target genes. LA might be a natural active compound candidate for breast cancer treatment.

## Graphical abstract

Effect of lobaric acid on Wnt/β‐catenin pathway in breast cancer.

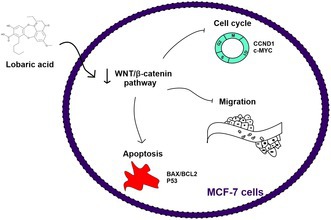

**Abbreviations**
APC: adenomatous polyposis coli
AXIN: axis inhibition protein
BAX: Bcl‐2–associated X protein
BCL2: Bcl2 apoptosis regulator
CCND1: cyclin D1
CDK1: cyclin‐dependent kinase 1
CK1: casein kinase 1
c‐MYC: MYC proto‐oncogene
DMEM: Dulbecco's modified eagle medium
DVL: disheveled
ER: estrogen
FBS: fetal bovine serum
FZD: frizzled
GSK‐3β: glycogen synthase kinase 3β
LA: lobaric acid
MCF‐7: human breast cancer cell line
P53: tumor protein p53
PR: progesterone
qPCR: quantitative real‐time PCR
TCF/LEF: T‐cell factor/Lymphoid enhancer factor

## 1 Introduction

Breast cancer is the most commonly diagnosed cancer among female patients and is the leading cause of cancer‐related deaths [1]. In 2023 estimates, there were 297 790 new cases of invasive breast cancer and 43 170 deaths in women in the United States [2]. According to the Global Cancer Observatory, the incidence of breast cancer in women around the world is estimated to reach 3.5 million cases by the year 2050. Breast cancer is divided into 3 main subtypes based on molecular markers for estrogen (ER) or progesterone (PR) receptors and human epidermal growth factor 2 (ERBB2; formerly HER2), and triple negative (tumors lacking all three standard molecular markers) [3]. Due to the complexity and heterogeneity of breast cancer, conventional treatment modalities have failed to provide effective treatment, leading to drug resistance and metastatic progression [4]. Thus, the increasing incidence of breast cancer emphasizes the need for new and effective approaches to breast cancer treatments with fewer side effects. Considering their low toxicity and ability to produce a wide range of metabolites, natural products and their derivatives are increasingly recognized as valuable sources for chemotherapeutic drugs [5, 6, 7, 8].

The search for new active pharmaceutical molecules derived from natural sources such as lichens is of great interest in the pharmaceutical industry. Lichens are known to grow on different surfaces in different regions and this is due to their ability to withstand extreme environments [9]. Lichens are formed by the symbiotic association of a species of fungus known as a mycobiont and a species of green algae known as a photobiont [10]. The mycobiont usually determines the morphology of the lichen and plays a role in its protection from adverse environmental factors, while the photobiont's main role is to synthesize organic compounds necessary for lichen growth. Lichen secondary metabolites, with a high degree of diversity, estimated at more than one thousand different compounds, are classified according to their chemical structure; phenolic compounds, pulvinic acid derivatives (e.g., vulpinic acid), quinones (e.g., parietin), aliphatic acids (e.g., protolichesterinic acid), dibenzofurans (e.g., usnic acid), and depsidones (e.g., salazinic acid, lobaric acid) [11]. These secondary metabolites can exhibit many biological activities, such as antimicrobial, antioxidant, antifungal, anti‐inflammatory, antimutagenic, and anticancer [12, 13, 14]. The potential of anticancer agents, particularly lichen metabolites, to impede metastasis and trigger apoptosis by suppressing cancer‐related signaling pathways represents a fascinating domain of research in cancer treatment.

The Wnt/β‐catenin signaling pathway has been implicated in many biological processes, including tumorigenesis, cell proliferation, cell cycle regulation, embryogenesis, metastasis, cellular differentiation, apoptosis, and drug resistance [15]. Wnt/β‐catenin is dysregulated and abnormally activated in cancer including breast cancer. This pathway is known to play an important role in certain breast cancer subtypes. Especially since two‐thirds of breast cancer patients are hormone receptor (HR^+^) dependent, the use of compounds that inhibit estrogen expression and antagonize the ER is considered a key element in endocrine therapy treatment [16]. Mutations and epigenetic modifications in components of the Wnt/β‐catenin signaling pathway, which is meticulously regulated under normal physiological conditions, can result in abnormal activation of the pathway and its downstream target genes. This process contributes to tumor formation and progression [17, 18]. This signaling pathway, involved in carrying out various biological functions, is initiated by Wnt binding to frizzled (FZD) and lipoprotein receptor‐related protein (LRP5/6) receptors. β‐catenin, the downstream effector in this cascade, serves as a vital component of the canonical Wnt pathway [19]. In the absence of Wnt binding, cytoplasmic β‐catenin undergoes phosphorylation by a degradation complex consisting of axis inhibition protein (AXIN), adenomatous polyposis coli (APC), glycogen synthase kinase 3β (GSK3β), E3 ubiquitin ligase β‐TrCP (SCFβ‐TrCP) and casein kinase 1α (CK1α). After phosphorylation of β‐catenin, ubiquitination and subsequent proteasomal degradation occur, and the target genes of this pathway cannot be activated [20]. In the presence of Wnt binding, disheveled (DVL) induction leads to complex aggregation at the receptor and phosphorylation and inhibition of GSK3β cause an increase in intracellular β‐catenin concentration [21]. After cytoplasmic accumulation, β‐catenin translocates to the nucleus and interacts with T‐cell factor/lymphoid enhancer factor (TCF/LEF) to induce transcriptional activation of its target genes [17]. In addition, the Wnt/β‐catenin pathway cross‐talks with many other signaling‐related or coordinated pathways such as the PI3K/Akt, Notch, Hippo/YAP signaling, EGFR, Sonic Hedgehog, and NF‐κB [22, 23, 24, 25]. Together, their bidirectional cross‐talk affects a number of key molecular pathways involved in cancer initiation and progression [26]. Therefore, inhibition of the Wnt/β‐catenin pathway in cancer cells has been highlighted as a potential target for cancer therapy as it suppresses proliferation, metastasis, and tumor progression [27, 28].

Lobaric acid, a secondary metabolite isolated from lichens such as *Stereocaulon*, *Usnea*, *Parmelia*, and *Cladonia*, has biological activities such as anti‐inflammatory, antimicrobial, antioxidant, enzyme inhibition, and cytotoxicity [29, 30, 31, 32, 33, 34]. There are a limited number of studies in the literature showing that LA affects proliferation and apoptosis in some cancer cells, but the interaction of LA with the Wnt/β‐catenin signaling pathway is not yet known [35, 36, 37]. In this study, it was to investigate the effect of LA on viability, migration, and apoptosis in human breast cancer MCF‐7 cells through its interaction with the Wnt/β‐catenin pathway.

## 2 Materials and Methods

### 2.1 Cell Culture and Treatment Conditions

The human breast cancer cell line (MCF‐7) was obtained from the American Type Culture Collection (ATCC). The cells were cultured in Dulbecco's Modified Eagle Medium (DMEM) (Gibco). This medium contained 1% penicillin/streptomycin (Sigma‐Aldrich), 1% L‐glutamine (Gibco), and 10% (v/v) heat‐inactivated fetal bovine serum (FBS) (HyClone), and the cells were cultured at 37°C in a 5% CO_2_ atmosphere.

### 2.2 Preparation of LA

LA (C_25_H_28_O_8_, purity of 96.3%) was isolated by ChromaDex (00012305) from the lichens *Stereocaulon* and *Parmelia*. LA was prepared as a stock solution (25 mg/mL) in dimethyl sulfoxide (DMSO, Sigma). It was stored at −20°C until needed.

### 2.3 Analysis of Cell Viability Using 2,3‐Bis‐(2‐Methoxy‐4‐Nitro‐5‐Sulphophenyl)‐2H‐Tetrazolium‐5‐Carboxanilide (XTT) Assay

Cells were cultured in medium at 37°C in 5% CO_2_ and seeded into 96‐well plates at a density of 1 × 10^4^ cells per well and allowed to attach to the plates overnight. LA was diluted with medium to final concentrations of 0, 10, 25, 37.5, 50, 75, and 100 μg/mL The cells were incubated with LA for 24 and 48 h. After incubation with the XTT assay (Cell Proliferation Kit, Biological Industries), absorbance was measured at 470 nm with an ELISA reader (BioTek, USA). IC_50_ values (50% inhibition concentration) were calculated using GraphPad Prism (version 5.0, Boston, MA, USA) [38].

### 2.4 Analysis of Cell Apoptosis Using Flow Cytometry

Cells were seeded at a density of 3 × 10^5^ cells/well in six‐well plates and incubated overnight at 37°C in a 5% CO_2_ atmosphere. LA‐treated MCF‐7 cells were incubated for 48 h. Following incubation, steps were performed using the Annexin V‐FITC/propidium iodide (PI) double staining detection kit according to the instruction manual (BioLegend, San Diego, CA). Sample detection was performed by flow cytometry (Beckman Coulter CytoFLEX, Brea, CA) [39].

### 2.5 Wound Healing Assay

A wound healing assay was applied to assess cell migration. Cells (5 × 10^5^ cells/well) were cultured in 6‐well plates. After the cells were 90% confluent, the scratch was created with a sterile 200 μL pipette tip. After washing with Dulbecco's phosphate‐buffered saline (DPBS) to remove cell debris, the cells were incubated in the medium supplemented with 5% FBS in the absence or presence of EA (44.21 μg/mL). The wound areas were photographed at 0, 6, 12, and 24 h via an inverted microscope. The migration distance was measured by the software program ImageJ. Results show the mean of six measurements of each wounded area, obtained in three independent experiments (*n* = 18) [6, 40].

### 2.6 Total mRNA Extraction and cDNA Synthesis

The MCF‐7 cell line was seeded at 2 mL (150.000 cells/mL) per well of a 6‐well plate and incubated at 37°C in a 5% CO_2_ atmosphere. The medium was then removed, and the wells were washed with DPBS (Gibco) buffer. Fresh complete medium was added to the control group. The treatment group was treated with LA (IC_50_ concentration at 48 h) determined in fresh complete medium in triplicate and cells were incubated at 37°C in a 5% CO_2_ atmosphere. The medium was aspirated and the wells were washed with DPBS. To harvest cells from the wells, 600 μL lysis buffer (10 μL β‐mercaptoethanol per 1 mL lysis buffer) was added to each well. RNA was isolated according to the manufacturer's protocol using the PureLink RNA Mini Kit (Invitrogen). The purity and concentrations of total RNA samples isolated from cells were determined by a microplate reader (Thermo Scientific Multiscan GO). RNA concentrations were equalized to ensure equal experimental conditions [41].

cDNA was synthesized from total RNA obtained from the cells using a high‐capacity cDNA reverse transcription kit (Applied Biosystems) according to the manufacturer's protocol. cDNA concentrations were equalized to ensure equal experimental conditions.

### 2.7 Analysis of Gene Expression by Quantitative Real‐Time PCR (qPCR)

The expression levels of *BAX, BCL2, P53, WNT2, DVL1, AXIN1, CTNNB1 (β‐catenin*), *TCF‐4, CCND1, c‐MYC*, and *CDK1* genes were measured by qPCR using SYBR Green Master Mix (BIORAD). *β‐Actin* was used as a housekeeping gene. To the qPCR reaction mixture; cDNA (100 ng–100 fg), 0.3–0.5 μM of each primer, 5 μL of Syber Green master mix (BioRad) were added, and the total volume was made up to 10 μL with nuclease‐free water. The reaction conditions were set to include a denaturation step at 95°C for 30 s followed by 40 cycles of 95°C for 5 s, ≤ 60°C for 30 s, and 72°C for 20 s. Table 1 shows the base sequences of the primers used in qPCR experiments. An analysis of the amplification curve was performed to measure the amplified products. Experiments were repeated in triplicate. Relative mRNA levels were calculated using the 2^−∆∆CT^ method [42].

**TABLE 1:** The list of primers used in this study.

Primers	Sequence (5′—3′)
*BAX*	Forward‐ CAGGAAGTGGAAAGGTGGAG
	Reverse‐ CTCAGGATGAAGACCCGAAG
*BCL2*	Forward‐ TTGTAGTGTGTATGCCCTGCTT
	Reverse‐ TCCTCTGTGATGCTGAAAGGT
*P53*	Forward‐ ACCCATCCACCTCTCATCAC
	Reverse‐ TCTACTCCCAACCACCCTTG
*WNT2*	Forward‐ AAGGAAAGGATGCCAGAGCC
	Reverse‐ TGCACATCCAGAGCTTCCAG
*DVL1*	Forward‐ GATGGACAACGAGACAGGCA
	Reverse‐ CGGCATCGTCATTGCTCATG
*AXIN1*	Forward‐ CGTCTGGAGGAGGAAGAAAAGAG
	Reverse‐ CTCTGCGATCTTGTCTCTGTCT
*CTNNB1 (β‐catenin)*	Forward‐ GCTTGGTTCACCAGTGGATT
	Reverse‐ GTTGAGCAAGGCAACCATTT
*TCF‐4*	Forward‐ GAGGCCAAGGTTTGTGTGAT
	Reverse‐ CACTGCTCACAGGAGGTGAA
*CDK1*	Forward‐ GGCTCTGATTGGCTGCTTTG
	Reverse‐ GGTAGATCCGCGCTAAAGGG
*c‐MYC*	Forward‐ TACAACACCCGAGCAAGGAC
	Reverse‐ GAGGCTGCTGGTTTTCCACT
*CCND1*	Forward‐ GGCGGAGGAGAACAAACAGA
	Reverse‐ CTCCTCAGGTTCAGGCCTTG
*β‐Actin*	Forward‐ TGCTATCCCTGTACGCCTCT
	Reverse‐ CTCCTTAATGTCACGCACGA

### 2.8 Protein Isolation and Western Blot Analysis

Cells were incubated with IC_50_ concentration of LA for 48 h and proteins (10 μg) from cells lysed with radioimmunoprecipitation assay buffer (RIPA, Cell Signaling Technology) were separated by 12% SDS‐PAGE. Subsequently, membrane incubation was performed overnight at 4°C using P53 antibody (Proteintech, 10442‐1‐AP, 1:1000), BCL2 antibody (Proteintech 12789‐1‐AP 1:500), WNT2 antibody (Proteintech, 66656‐I‐Ig, 1:1000), AXIN antibody (Proteintech, 68093‐1‐Ig, 1:1000), β‐Catenin antibody (Santa Cruz Biotechnology, sc‐7963, 1:1000), GSK3‐ β antibody (Santa Cruz, sc‐377213, 1:1000), and β‐Actin antibody (Santa Cruz Biotechnology, sc‐47778, 1:1000). Protein samples were visualized after application of horseradish‐linked secondary antibodies (Santa Cruz Biotechnology) at 1:10 000 dilution and exposure to a chemiluminescence detection system (ECL Clarity/ECL Clarity Max Substrate, Biorad). Bands were measured with ImageJ2x and relative intensities were calculated based on the intensity of β‐actin bands in each sample [43].

### 2.9 Statistical Analysis

Data are presented as mean ± SEM from three experiments. The unpaired t‐test and one‐way analysis of variance (ANOVA) were performed to statistically compare the results in GraphPad Prism. An asterisk (*) indicates statistically significant changes. Symbols are defined below: *p* > 0.05 (not significant, ns); *p < 0.05 (significant), **p < 0.01 (highly significant), and ***p < 0.001 (extremely significant).

## 3 Results

### 3.1 LA Significantly Inhibits Breast Cancer Cell Viability

**FIGURE 1:**
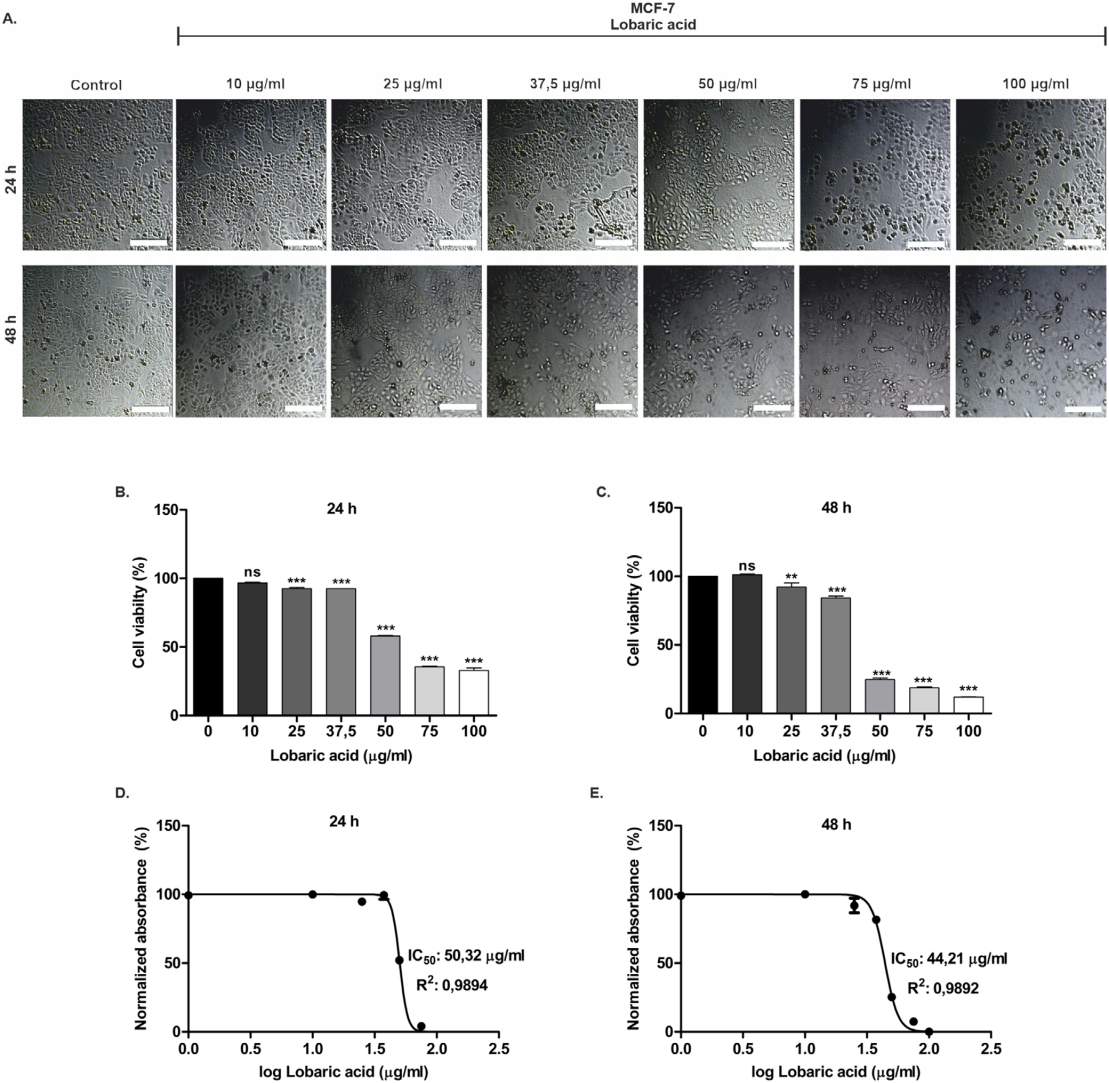
Inhibition of MCF‐7 cell viability by LA. (A) Dose (10, 25, 37.5, 50, 75, and 100 μg/mL) and time (24 and 48 h) dependent inverted microscope images of MCF‐7 cells treated with LA. (B, C) MCF‐7 cells were treated with different LA concentrations for 24 and 48 h and cell viability was determined by XTT assay. (D, E) IC_50_ values were calculated using GraphPad Prism. The experiment was performed in three biological and technical replicates. **p < 0.01, and ***p < 0.001 in relation to the control. Scale bar, 500 μm.

The effects of LA on MCF‐7 cell viability were investigated in vitro using the XTT assay. For this purpose, MCF‐7 cells were exposed to 0–100 μg/mL concentrations of LA in a time‐dependent manner (24 and 48 h) to evaluate its cytotoxic effect. As indicated in Figure 1A, the cells showed morphological changes at increasing doses of LA. In MCF‐7 cells, LA had a cytotoxic effect at doses of 25 μg/mL and above at both times (Figure 1B,C). In LA‐induced cells, the IC_50_ value was calculated as 50.32 ± 0.84 μg/mL at 24 h and 44.21 ± 1.1 μg/mL at 48 h (Figure 1D,E). The findings showed that LA significantly decreased the viability of MCF‐7 cells.

### 3.2 LA Induces Apoptosis in MCF‐7 Cells

**FIGURE 2:**
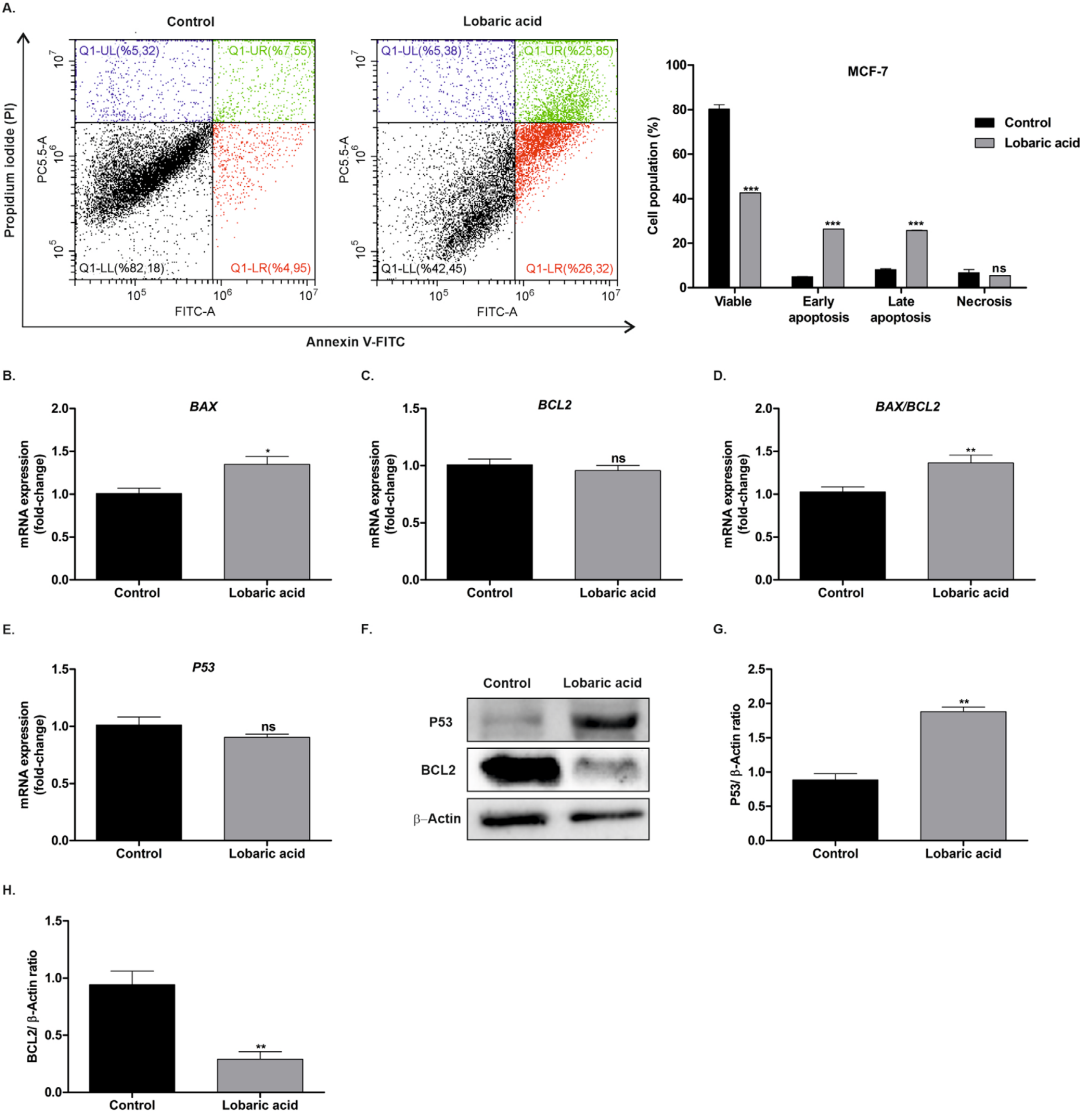
Induction of apoptosis of MCF‐7 cells by LA. Flow cytometry was performed to determine the pro‐apoptotic effect of LA (IC_50_ concentration at 48 h) on MCF‐7 cells. (A) Cell population distributions were determined after treatment with LA at 48 h. Top left identifies necrotic cells (PI single positive); top right late apoptotic cells (annexin V and PI double positive); bottom left early apoptotic cells (annexin V single positive) and bottom right viable cells (non‐apoptotic cells). (B–E) *BAX*, *BCL2*, and *P53* mRNA levels and the BAX/BCL2 ratio were analyzed by qPCR in MCF‐7 cells. (F–H) P53 and BCL2 protein expressions were determined by Western blot analysis. The experiment was performed in three biological and technical replicates. *p < 0.05, **p < 0.01, and ***p < 0.001 in relation to the control.

To observe the apoptotic and necrotic effects of LA, MCF‐7 cells were treated with its IC_50_ concentration for 48 h and subsequently stained with Annexin‐FITC/PI (Figure 2A). While the viable cell population was 80.2% ± 2.8% in the control group, it decreased to 42.6% ± 0.2% in the LA‐treated group (*p* < 0.001). Furthermore, the early and late apoptotic cell populations in the control group were 5.0% ± 0.1% and 8.1% ± 0.7%, respectively, but increased to 26.3% ± 0.1% and 25.7% ± 0.2% in the LA‐treated group (*p* < 0.001). The necrotic cell population was 6.7% ± 2.0% in the control group and 5.4% ± 0.1% in the treatment group, but this was not statistically significant. Additionally, qPCR analysis was performed to evaluate the effect of LA on apoptosis at the molecular level. The findings indicated an increase in *BAX* gene expression (*p* < 0.05), a pro‐apoptotic marker, and a decrease in *BCL2* gene expression (*p* > 0.05), an anti‐apoptotic marker (Figure 2B,C). Thus, an increase in the BAX/BCL2 ratio (*p* < 0.01), which is an important parameter for apoptosis, was observed (Figure 2D). The expression levels of the *P53* gene, which functions as a distinct apoptotic marker, did not exhibit any significant statistical discrepancy. However, Western blot analysis revealed a substantial augmentation in P53 (*p* < 0.01) protein levels and a decrease in BCL2 (*p* < 0.01) protein levels (Figure 2G,H). These results confirmed that LA inhibited MCF‐7 cell proliferation by inducing apoptosis.

### 3.3 LA Suppresses Migration by Inhibiting the Wnt/β‐Catenin Pathway in MCF‐7 Cells

The effect of LA on tumor migration, which is the basic step in tumor formation, was determined by wound healing analysis. For the wound healing assay, images were taken at different times (0, 6, 12, and 24 h) after LA treatment at IC_50_ concentration in MCF‐7 cells, and then wound areas were analyzed. In MCF‐7 cells, the percentage of wound closure in the control group at 6, 12, and 24 h compared to 0 h was approximately 26%, 30%, and 39%, respectively, while in the LA‐treated group it was approximately 11%, 5%, and 2%, respectively, at the same time points. These results indicate that wound closure was faster in the control group cells compared to LA.

**FIGURE 3:**
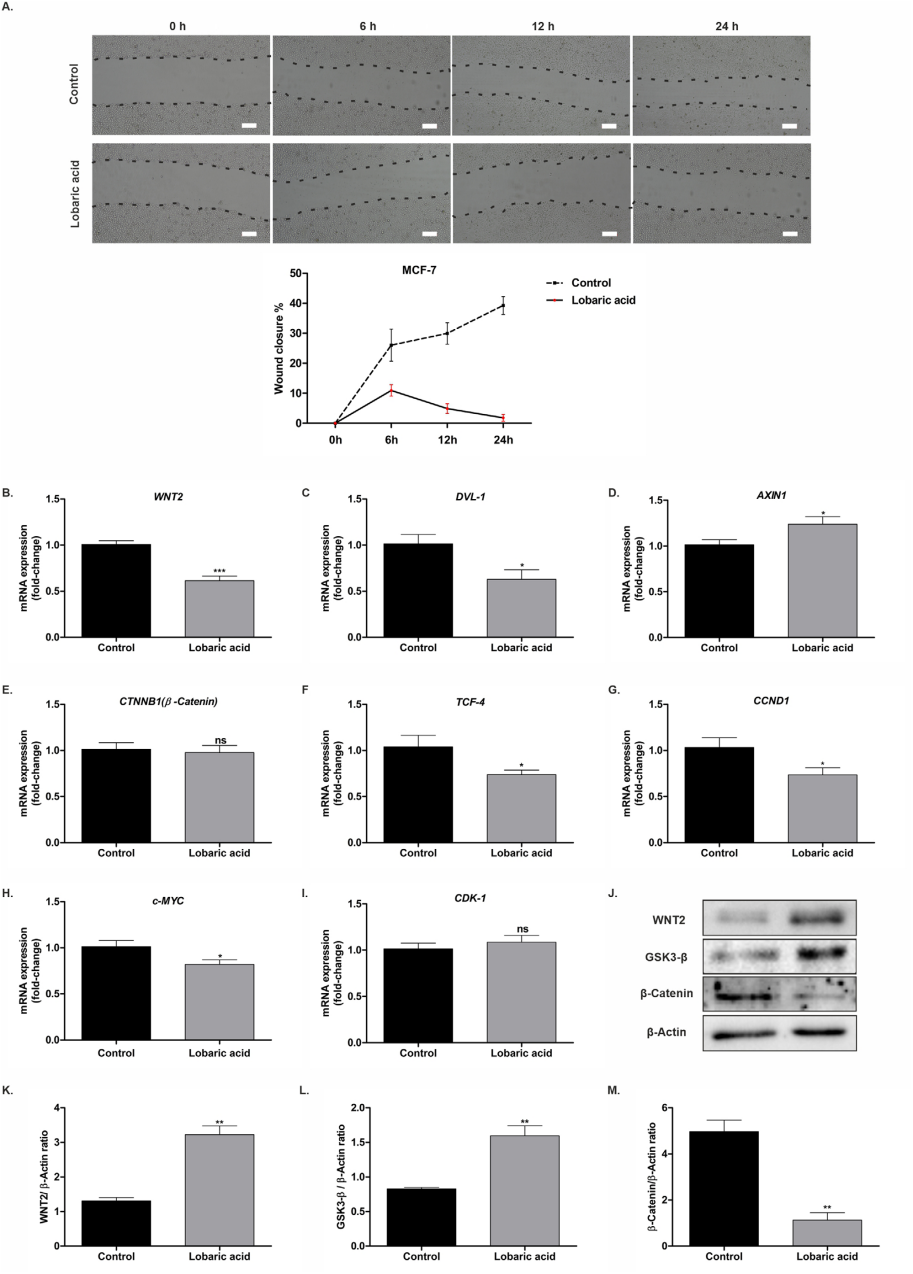
Effect of LA on Wnt/β‐catenin pathway in MCF‐7 cells. (A) The migration of cells treated with LA into the wound was visualized at 0, 6, 12, and 24 h under an inverted microscope, and the percentage of wound closure was evaluated statistically. (B–I) Expressions of *WNT2*, *DVL1*, *AXIN1*, *β‐Catenin*, *TCF‐4*, *CCND1*, *c‐MYC*, and *CDK1* genes analyzed by qPCR in LA‐treated MCF‐7 cells at 48 h. (J–M) Protein expressions of WNT2, GSK3‐ β, and β‐catenin determined by Western blot analysis. The experiment was performed in three biological and technical replicates. *p < 0.05, **p < 0.01, and ***p < 0.001 in relation to the control. Scale bar, 500 μm.

The potential effect of LA on the Wnt/β‐catenin signaling pathway, which plays important roles in tumorigenesis and metastasis, was investigated by qPCR and Western blot analysis. According to qPCR results, a significant decrease in *WNT2* (*p* < 0.001), *DVL‐1* (*p* < 0.05), and *TCF‐4* (*p* < 0.05) mRNA levels, but an increase in *AXIN1* (*p* < 0.05) was observed in LA‐treated MCF‐7 cells. However, there was no significant statistical difference in *β‐catenin* gene expression (*p* > 0.05) (Figure 3B–F). When *CCND1*, *c‐MYC*, and *CDK1* genes, which are Wnt/β‐catenin pathway target genes, were evaluated after LA treatment in MCF‐7 cells, it was observed that *CCND1* (*p* < 0.05) and *c‐MYC* (*p* < 0.05) were considerably suppressed. However, *CDK1* gene expression (*p* > 0.05) was not affected (Figure 3G–I). The results of the Western blot analysis demonstrated that LA increased WNT2 (*p* < 0.01) and GSK3‐β (*p* < 0.01) protein expressions, while also decreasing β‐catenin (*p* < 0.01) levels in MCF‐7 cells. (Figure 3J–M). These findings confirmed that LA suppresses the migration of MCF‐7 cells through the Wnt/β‐catenin pathway.

## 4 Discussion

Cancer is a significant health concern that poses a threat to human life [44]. Despite the various treatment methods employed, there are still some risks that remain unmitigated. Natural compounds have gained significant popularity in cancer treatment due to their minimal adverse effects, numerous therapeutic benefits, and ease of acquisition [45]. Lichens, a natural compound, have been used in medical treatments since time immemorial [46]. Especially the metabolites produced by lichens are known to have antiproliferative, apoptotic, anti‐metastatic, and anti‐angiogenic properties [9]. The anti‐tumor activities of lichen secondary metabolites in several cancer cells such as breast, gastric, colorectal, liver, and lung have been reported in the literature [8, 47, 48, 49, 50]. Hence, this study was designed to investigate the antiproliferative, apoptotic, and antimigratory potential of LA, a secondary metabolite of lichens, on the breast cancer MCF‐7 cell line.

Anti‐proliferation tests are frequently utilized to ascertain the potential cytotoxic effect of a synthetic or natural compound on cells. In this study, the findings indicated that LA exhibited a significant suppression effect on the cell viability of MCF‐7 cells, with an IC_50_ value of 44.21 μg/mL (equal to 96.8 μM) at 48 h. In the literature, Hong et al. [35], reported that LA inhibited the proliferation of human cervical adenocarcinoma HeLa cells and colon carcinoma HCT116 cells in a dose‐ and time‐dependent manner, and the effective IC_50_ value was calculated as 50 μM in both cell lines. Emsen et al. [37] showed that the viability of glioblastoma multiforme (GBM) cells and primary rat cerebral cortex (PRCC) cells was significantly reduced by LA treatment for 48 h and IC_50_ values were determined as 5.77 and 9.08 mg/L, respectively. Kizil et al. [36] revealed that different doses of LA (12.5, 25, 50, and 100 μg/mL) had a very slight effect on the viability of human lung cancer A549 cells. Kwon et al. [51] examined the antiproliferative effect of LA on mouse vascular smooth muscle cell line (MOVAS‐1) for 24 h and showed that cell growth was inhibited at 100 μg/mL. Brisdelli et al. [52] reported that LA had an antiproliferative effect in MCF‐7, HeLa, and HCT‐116 cell lines and IC_50_ values were calculated as > 100, 78.0, and 93.2 μM, respectively. In our previous study, the cytotoxic effects of carboplatin and docetaxel, which are commercial anticancer drugs, were determined as 33.35 μg/mL (equal to 89.8 μM) and 64.32 μg/mL (equal to 79.6 μM), respectively, on MCF‐7 cells [43]. The present data showed that LA has a cytotoxic effect close to that of commercial anticancer drugs.

Apoptosis, a process of cell death, is a critical component of cellular homeostasis in healthy tissues. However, cancer cells often exhibit resistance to apoptosis, leading to uncontrolled proliferation [53]. Therefore, induction of apoptosis is an effective strategy in cancer therapies. BAX (pro‐apoptotic) and BCL2 (anti‐apoptotic), members of the mitochondrial apoptotic pathway, are regulated during carcinogenesis and prevent the death of cancer cells [54]. The upregulation in BAX expression, but downregulation in BCL2 expression in cancer therapies may indicate that cells are directed to apoptosis. Therefore, an elevated BAX/BCL2 ratio serves as an indicator of apoptosis [55]. Moreover, P53 plays a critical role in tumor suppression by inducing apoptosis, growth arrest, and senescence, and it is one of the attractive targets for anticancer drug discovery due to its suppression in cancer cells [56]. At this stage of the study, the findings showed that although there was surprisingly no change in *BCL2* gene expression in LA‐treated MCF‐7 cells, the BAX/BCL2 ratio was increased due to increased *BAX* gene expression. Furthermore, the increase in P53 protein expression and decrease in BCL2 protein expression confirmed that apoptosis was induced. Moreover, the fact that LA considerably induced apoptotic cell death but not necrosis in these cells confirmed our gene and protein expression results. Our results were supported by the literature. For instance, it was reported that LA increased BAX gene expression in A549 cells at a concentration of 100 μg/mL [36]. Another study reported that LA dose‐dependently increased the apoptotic cell population in HeLa cells [35].

Metastasis is one of the leading causes of cancer‐related deaths, and metabolic processes, tumor microenvironment, metastasis‐related genes and important signaling pathways, etc. need to be investigated to prevent metastasis in cancer cells [57]. In this study, LA was found to have a significant antimigratory effect in MCF‐7 cells by the wound healing assay. In this regard, to determine the antimigratory effect of LA‐treated cells at the molecular level, we aimed to elucidate the Wnt/β‐catenin signaling pathway at the gene and protein levels, as it is involved in many biological processes, including tumorigenesis, cell cycle regulation, apoptosis, and metastasis. In the literature, natural compounds including some lichen secondary metabolites have been reported to show anticancer effects by inhibiting Wnt signaling in glioblastoma multiforme, lung cancer, and colorectal cancer [8, 49, 58, 59]. It has been reported in the literature that WNT2 is overexpressed in colorectal cancer, cervical cancer, human fibroadenomas, pancreatic cancer, and breast cancer, thereby triggering migration and invasion [60, 61, 62, 63, 64]. The observation that LA suppresses the mRNA level of *WNT2* in MCF‐7 cells, which plays an important role in the activation of this pathway, is an important parameter for its inactivation. Contrary to our initial expectation, the increase in WNT2 protein expression was observed in LA‐treated MCF‐7 cells, in contrast to its gene expression level. Numerous studies have previously investigated the relationship between mRNA and protein expression in different human tissues. Surprisingly, these have revealed that strong correlations are lacking, such that mRNA and protein correlations are reported to be 20%–40% [65, 66, 67, 68, 69]. It has been observed that transcripts and proteins exhibit variability in their correlation, both across tissues (within‐gene correlation) and across genes (across‐gene correlation). It is hypothesized that this variability is the result of temporal mRNA dynamics, post‐transcriptional and post‐translational regulation, translation rates, protein transport and half‐lives, and protein synthesis constraints and delays [70, 71, 72]. Furthermore, protein stability is an important factor in this process, i.e., the half‐life of different proteins ranges from minutes to days, while the rate of mRNA degradation is much shorter [73]. Given that AXIN1 negatively regulates the signaling pathway, the substantial increase in *AXIN1* expression in LA‐induced MCF‐7 cells may be indicative of the activation of the destruction complex [74]. As for DVL‐1, which is highly expressed in various tumor types including breast cancer, it is targeted for suppression in cancer therapies because it regulates oncogenic Wnt signaling, an important driver of tumor progression [75]. It was shown that LA had a significant effect on suppressing *DVL‐1* mRNA levels in MCF‐7 cells. In the Wnt signaling pathway, GSK3‐β is an essential protein that controls β‐catenin [76]. In the absence of Wnt signaling, activated GSK3‐β phosphorylates β‐catenin, resulting in its degradation [77, 78]. The results of the Western blot assay demonstrated that LA‐induced GSK3‐β protein in MCF‐7 cells. Regarding the levels of *β‐catenin* mRNA, a trend toward downregulation was observed in LA‐treated cells, though this did not reach statistical significance. However, a significant decrease in β‐catenin protein was observed in MCF‐7 cells. It is noteworthy that the increase in GSK3‐β may have resulted in the degradation of β‐catenin. In literature, Ko et al. treated leukemia U937 cells with cordycepin (an active ingredient of traditional medicine) alone or in combination with SB216763, a pharmacological inhibitor of GSK3‐β. They observed that β‐catenin reduced by cordycepin was significantly restored by SB216763. This suggests that degradation of β‐catenin is mediated by the regulation of GSK3‐β. Furthermore, cordycepin significantly inhibited Akt (Ser473) phosphorylation, which is associated with the degradation of β‐catenin, while it decreased Akt and GSK3‐β (Ser9) phosphorylation in a time‐dependent manner. These results suggest that cordycepin may regulate GSK3‐β by inactivating PI3‐K/Akt signaling, thereby inducing protein degradation of β‐catenin [79]. Since our results showed an increase in WNT2 protein, it is also hypothesized that LA may regulate GSK3‐β, which is involved in the inhibition of β‐catenin through PI3‐K/Akt signaling. Our results may indicate that inhibition of β‐catenin may play a role in the suppression of *TCF‐4* gene expression. Thus, *c‐MYC* and *CCND1*, which are target genes of this pathway, were suppressed in LA‐treated MCF‐7 cells. It is noteworthy that the repression of these target genes is known to be critical in regulating cell cycle transitions and fostering oncogenesis. Therefore, the observed decrease in their expression serves to augment the anticancer effect of LA in MCF‐7 cells.

## 5 Conclusions

In summary, the present study demonstrated for the first time that LA suppresses cell viability and migration, as well as induces apoptosis by modulating the Wnt/β‐catenin pathway in MCF‐7 cells. Thus, LA may be a potential anticancer agent for the treatment of breast cancer through the Wnt/β‐catenin pathway.

## Author Contributions

**Şeyda Nur Kalın:** conceptualization, data curation, formal analysis, investigation, methodology, validation, visualization, writing – original draft, writing – review and editing. **Kübra Nur Bayındırlı:** data curation, formal analysis, investigation, methodology, validation. **Emine Toraman:** data curation, formal analysis, investigation, methodology, validation. **Şükran Günaydın:** investigation, methodology, validation. **Fatmanur Keleş:** investigation, methodology, validation. **Ahmet Altay:** conceptualization, project administration, resources, supervision, validation, writing – review and editing. **Harun Budak:** conceptualization, project administration, resources, supervision, validation, writing – review and editing. The final version of the manuscript was read and approved by all authors.

## Ethics Statement

The authors have nothing to report.

## Conflicts of Interest

The authors declare no conflicts of interest.

## Data Availability

The data that support the findings of this study are available from the corresponding author upon reasonable request.
